# Quality of Life and Associated Socio-Clinical Factors after Encephalitis in Children and Adults in England: A Population-Based, Prospective Cohort Study

**DOI:** 10.1371/journal.pone.0103496

**Published:** 2014-07-29

**Authors:** Parashar Pravin Ramanuj, Julia Granerød, Nicholas W. S. Davies, Stefano Conti, David W. G. Brown, Natasha S. Crowcroft

**Affiliations:** 1 Reference Microbiology Service, Public Health England, London, South London, United Kingdom; and Maudsley NHS Foundation Trust, London, United Kingdom; 2 Virus Reference Department, Public Health England, London, United Kingdom; 3 Department of Neurology, Chelsea & Westminster Hospital, London, United Kingdom; 4 Statistics, Modelling and Economics Department, Public Health England, London, United Kingdom; 5 Public Health Ontario, Ontario, Canada; Cardiff University, United Kingdom

## Abstract

**Objective:**

We sought to measure HRQoL in all-cause encephalitis survivors and assess the impact of various socio-clinical factors on outcome.

**Methods:**

We used a prospective cohort study design, using the short-form 36 (SF-36) to measure the HRQoL in patients 15 years and older, and the short-form 10 (SF-10) for patients less than 15 years old. We posted questionnaires to individuals six months after discharge from hospital. All scores were normalised to the age- and sex-matched general population. We used multivariate statistical analysis to assess the relative association of clinical and socio-demographic variables on HRQoL in adults.

**Results:**

Of 109 individuals followed-up, we received 61 SF-36 and twenty SF-10 questionnaires (response rate 74%). Patients scored consistently worse than the general population in all domains of the SF-36 and SF-10, although there was variation in individual scores. Infectious encephalitis was associated with the worst HRQoL in those aged 15 years and over, scoring on average 5.64 points less than immune-mediated encephalitis (95% CI −8.77– −2.89). In those aged less than 15 years the worst quality of life followed encephalitis of unknown cause. Immuno compromise, unemployment, and the 35–44 age group all had an independent negative association with HRQoL. A poor Glasgow Outcome Score was most strongly associated with a poor HRQoL. Less than half of those who had made a ‘good’ recovery on the score reported a HRQoL equivalent to the general population.

**Conclusions:**

Encephalitis has adverse effects on the majority of survivors’ wellbeing and quality of life. Many of these adverse consequences could be minimised by prompt identification and treatment, and with better rehabilitation and support for survivors.

## Introduction

Encephalitis is a potentially life-threatening neurological syndrome characterised by inflammation of the brain parenchyma. It can be caused by infection or immune-mediated conditions. Its incidence in England has recently been estimated to be 5.23/100,000/year (but it could be as high as 8.66/100,000/year) [1]. Mortality is thought to be about 12% [2].

The high morbidity and mortality associated with the illness result from inflammatory processes caused by microbial neuro virulence or direct immune-mediated damage. For survivors, the consequences of encephalitis can be severe. Three years after infectious encephalitis 51.7% seek help from general practitioners, for one or more symptoms [3]. The most common problems are concentration difficulties (42%), behavioural disorders (27%), speech disorders (20%) and memory loss (19%) [3].

What is less clear is the quality of life of patients who survive encephalitis. It is likely these individuals suffer subtle problems that are difficult to quantify. Research has concentrated on the long-term neurocognitive effects of encephalitis but the consequences for patients and their families have largely been neglected. The ability to measure the impact of a disease according to a patient’s perspective is important in identifying foci for treatment delivery as well as further research directions [4], [5].

Our aim was to establish the health-related quality of life (HRQoL) in patients who survived an episode of encephalitis and compare this with a normative population. We identified any differences in HRQoL by aetiological category and assessed the effect of socio-clinical factors on post-encephalitic HRQoL.

## Methods

### Patients

These analyses were built on a previous multi-centre, population-based, prospective study of encephalitis in England. We have reported details regarding recruitment and selection criteria elsewhere [2]. In summary, patients were recruited over two years from 24 participating centres in England; there was a staged start from October 2005 to November 2006. A stringent case definition was used to define encephalitis. Briefly, any person of any age admitted to hospital within the centres selected during the recruitment period with encephalopathy (altered consciousness that persisted longer than 24 hours with lethargy, irritability or a change in character or behaviour) and with at least two of the following features: core body temperature ≥38°C or history of fever during the presenting illness; seizures and/or focal neurological findings; more than four white blood cells per millilitre in the cerebrospinal fluid; EEG findings indicative of encephalitis; and abnormal results of neurological CT or MRI scans suggestive of encephalitis were included in the original study. Of these, all patients aged ≥5 years discharged from hospital were eligible for our HRQoL study.

The North and East Devon Multicentre Research Ethics Committee granted overall approval for the study (05/Q2102/22). Local research ethics committee approval and Research and Development approval was also gained from all participating centres (Table 1). We obtained written informed consent from all patients or their next of kin.

**Table 1:** Local Research Ethics Committees and Research from the participating centres which granted approval for the study to be carried out.

Central Manchester Local Research Ethics Committee
Cumbria and Lancashire B
East London & City HA Local Research Ethics Committee
Institute of Child Health/Great Ormond Street Hospital Research Ethics Committee
The Joint UCL/UCLH Committees on the Ethics of Human Research (Committee Alpha)
King’s College Hospital Research Ethics Committee
Liverpool Paediatric Research Ethics Committee
The National Hospital for Neurology and Neurosurgery & Institute of Neurology Joint Research Ethics Committee
North Manchester Local Research Ethics Committee
Salford & Trafford Local Research Ethics Committee
Sefton Local Research Ethics Committee
South Devon Research Ethics Committee
South West Devon Research Ethics Committee
St Thomas’ Hospital Research Ethics Committee
Wandsworth Local Research Ethics Committee

### Procedure

We used the Short Form 36-item survey (SF-36) version 2 to measure HRQoL in individuals aged ≥15 years and the Short Form 10-item (SF-10) Health Survey for Children in those aged 5–14 years [6], [7]. The questionnaires were administered by post three-six months after hospital discharge. Postal administration of these questionnaires has been validated [8]. We followed any outstanding responses by telephone.

The SF-36 is one of the most widely evaluated generic instruments of HRQoL, with demonstrably good validity and internal consistency [8]. It measures eight important health-related domains: physical functioning (PF), role limitations caused by physical dysfunction (RP), bodily pain (BP), general health perceptions (GH), vitality (VT), social functioning (SF), role limitations caused by emotional difficulties (RE) and mental health perceptions (MH) [6]. We used the second version of the survey (SF-36) as it improves the range and precision of the role functioning scales [9].

Responses to the survey were transformed into norm based scores (NBS) as described in the scoring manual [9]. These were age- and sex-matched against a normative UK population using the Oxford Health Life Survey III dataset [10]. The resulting standardised scores have a mean value of 50 and a standard deviation of 10 points, so any value less than 50 is suggestive of a worse outcome compared to the general population. As this normative dataset included individuals aged 18–65 years, 12 subjects in our SF-36 group aged more than 65 years were matched to the nearest age group (55–65 years). Data from these 12 individuals were excluded in subsequent sensitivity analyses to assess their effect on SF-36 scores.

The SF-10 is a 10-item questionnaire designed to measure HRQoL in children. Unlike the SF-36 it should be completed by caregivers. The scoring method yields two summary measures: a physical summary score (PHS) and a psychosocial summary score (PSS), which are also norm-based and fluctuate around a mean of 50 with a standard deviation of 10 [7].

### Analysis

Study variables included patient demographics, occupation, immune competency, co-morbid illness and length of hospital stay. We categorised aetiologies as infectious, immune-mediated and unknown. We used occupation to indicate socio-economic status as defined by the National Statistics Socio-Economic Classification (NS-SEC) Three Class Categorisation [11]. We used the occupation of the patient if he/she was of working age, and that of the primary caregiver if the patient was not of working age to determine socio-economic status. Outcome at six months after discharge from hospital was scored according to the Glasgow Outcome Score (GOS), which divides clinical outcome into five objective categories: good recovery, moderate disability, severe disability, vegetative state and dead [12]. This provides an objective, clinician-rated measure of recovery at six months as opposed to the necessarily subjective measures of HRQoL as scored by the SF-36 and Sf-10.

We compared the demographic and clinical characteristics 1) across the three aetiological groups for individuals that completed the SF-36 and 2) between individuals that completed the HRQoL questionnaires and those that did not complete. A comparison of variables by a etiology was not possible for the SF-10 due to the small sample size. We carried out the descriptive data analysis using Stata v12 (Stata Corp, College Station, Texas). Differences in proportions were assessed by Fisher’s exact or Chi-Squared test as appropriate. The nonparametric equality-of-medians test was used to assess any difference in medians.

We carried out a multivariate regression analysis of SF-36 scores with exploration for pair-wise interactions to identify socio-demographic and prognostic factors influencing post-recovery HRQoL. We addressed incomplete responses using extrapolation techniques as laid out in the scoring manual [9]. Model fitting, selection and validation were implemented in a Bayesian framework via the simulation-based JAGS platform within the R statistical environment (results not shown) [13], [14].

We categorised HRQoL as ‘good’, ‘moderate’ or ‘poor’ based on the scoring manual recommendation that ‘group mean scores below 47 can be interpreted as being below the average range for the general population… [where] the standard deviations for each scale are equalized at 10′ [9]. We defined a ‘good’ HRQoL as a norm-based score that was equivalent to the general population mean (i.e. a score of 47 or more). A moderate HRQoL was a score one standard deviation less than good (i.e. a score between 37 and 46.9). A ‘poor’ HRQoL was a score less than 37. We compared the proportions of patients reporting HRQoL in each category with aetiology and outcome as measured by the GOS (good recovery, moderate disability and severe disability). Fisher’s exact test with the Freeman-Halton extension was used to compare the differences in proportions between good, moderate and poor HRQoL in each GOS outcome and aetiological group. The time of GOS assessment correspond edroughly to the median time to completion for the HRQoL measures.

## Results

**Figure 1:**
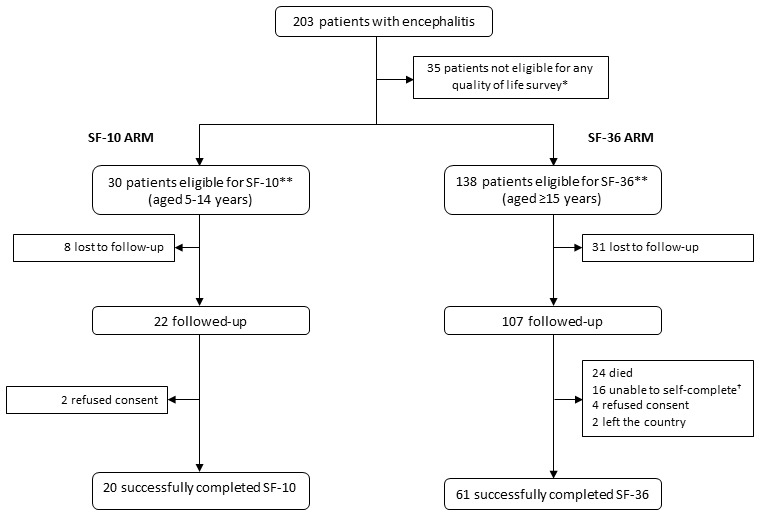
Study Profile. * These patients were 4 years old or younger and no appropriate tool could be found to appropriately assess their HRQoL. ** One 14 year-old patient completed the SF-36 and two completed the SF-10. † These patients were unable to self-complete as their functioning following encephalitis was so significantly impaired.

203 patients were identified with encephalitis in the initial prospective study. Of these, 55 were ineligible for either measure because they died pre-discharge (N = 20) or were less than 5 years old (N = 35) (Figure 1). We followed-up 87 of the 118 patients eligible for the SF-36 (74%) and 22 of the 30 eligible for the SF-10 (73%). From these, 61 complete SF-36 and 20 SF-10 questionnaires were ultimately received (4 died post-discharge, 16 were too impaired to self-complete the SF-36, 6 refused consent, 2 had left the country). Median time to completion was 5.6 months (range: 3–22 months).

### Baseline Characteristics

A definitive aetiology was identified in 41 (67.2%) individuals who completed the SF-36. Of these, 31 (75.6%) had an infectious cause (14 Herpes simplex virus (HSV), 6 *Mycobacterium tuberculosis,* 4 other bacterial, 4 Varicella zoster virus (VZV), 1 Epstein-Barr virus, 1 measles virus – sclerosing subacute panencephalitis, 1 dual infection with bacteria and fungi) and 10 (24.4%) were immune-mediated (2 acute disseminated encephalomyelitis (ADEM), 7 vantibody-associated, 1 multiple-sclerosis associated).

Adult patients with immune-mediated encephalitis had a significantly longer duration of hospital stay than those with an infectious cause (56.0 vs 30.5 days, p = 0.03). There was little evidence patients differed by aetiology in terms of any other demographic or clinical characteristics (Table 2). Of 63 patients with less-than-good recovery at discharge as determined by the GOS, patients who returned completed forms were more likely to be moderately disabled (34.4% vs. 17.5%, p = 0.04) and less likely to be severely disabled (18.1% vs. 36.8%, p = 0.02) than those who did not respond. Four patients who did not respond had died. Adult patients that were eligible but did not complete the SF-36 were more likely to be African-Caribbean or Black British (p = 0.02) and less likely to have a higher managerial or professional occupation (p = 0.04). The aetiological make-up of encephalitis in those that completed the SF-36 did not differ statistically from those that did not complete.

**Table 2:** Sample Characteristics in those Eligible for the SF-36.

SF-36		Aetiological Category					
	Infectious^a^(N = 31)	Immune-mediated^a^(N = 10)	Unknown(N = 20)	p	Completed theSF-36 (N = 61)	Did not complete theSF-36 (N = 57)	p
**Age, median (IQR), y**	54.0 (36.0–65.0)	36.5 (18.5–59.5)	54.0 (37.0–62.0)	0.457	52.0 (32.0–63.0)	40.0 (28.0–56.0)	0.213
**No. (%) male**	17 (54.8)	6 (60.0)	12 (60.0)	0.920	35 (57.4)	28 (49.1)	0.369
**Ethnicity, n (%)**							
White British or White Other	23 (74.2)	7 (70.0)	18 (90.0)	0.309	48 (78.7)	39 (68.4)	0.205
Asian or Asian British	4 (12.8)	0 (0.0)	1 (5.0)	0.354	5 (8.1)	3 (5.3)	0.718
African-Caribbean or Black British	2 (6.5)	1 (10.0)	1 (5.0)	0.872	**4 (6.6)**	**13 (22.8)**	**0.017**
Other (inc. Chinese and Mixed Ethnicity)	2 (6.5)	2 (20.0)	0 (0.0)	0.113	4 (6.6)	2 (3.5)	0.680
**NS-SEC Three Class Categorisation at admission, n (%)** b							
Higher managerial and professional occupations	7 (22.5)	2 (20.0)	4 (20.0)	0.970	**13 (21.3)**	**4 (7.0)**	**0.036**
Intermediate occupations	7 (22.5)	2 (20.0)	5 (25.0)	0.952	14 (22.9)	13 (22.8)	0.985
Routine and manual occupations	7 (22.5)	1 (10.0)	6 (30.0)	0.469	14 (22.9)	12 (21.1)	0.804
Never worked or long-term unemployed	2 (6.5)	1 (10.0)	2 (10.0)	0.880	5 (8.2)	5 (8.8)	1.000
Not classifiable (retired, students, etc)	8 (26.0)	4 (40.0)	3 (15.0)	0.317	15 (24.6)	23 (40.3)	0.067
**No. (%) immunocompromised**	4 (12.8)	0 (0.0)	2 (10.0)	0.492	**6 (9.8)**	**14 (24.6)**	**0.033**
**No. (%) with at least one co-morbidity** c	17 (54.8)	8 (80.0)	13 (65.0)	0.456	**38 (62.3)**	**45 (78.9)**	**0.048**
**Glasgow Outcome State 6 months after discharge, n (%)**							
Good recovery	14 (45.0)	3 (30.0)	12 (60.0)	0.280	29 (47.5)	22 (38.6)	0.327
Moderate disability	12 (38.7)	6 (60.0)	3 (15.0)	0.059	21 (34.4)*	10 (17.5)*	**0.037**
Severe disability	5 (16.3)	1 (10.0)	5 (25.0)	0.557	11 (18.1)*	21 (36.8)*	**0.022**
Dead	–	–	–	–	0	4 (7.1)	0.052
**Length of stay, median (IQR), d**	**30.5 (19.5–57.0)**	**56.0 (26.5–116.0)**	**20.5 (14.0–31.0)**	**0.029**	28.5 (16.0–44.0)	35.0 (19.5–100.5)	0.089

a Infectious causes included viral (Herpes simplex, Varicella zoster, measles and Epstein-Barr virus), bacterial (predominantly *Mycobacterium tuberculosis*) and dual bacterial-fungal infection. Immune-mediated causes included those associated with N-methyl-D-aspartate-receptor antibodies, voltage-gated potassium channel-complex antibodies, acute disseminated encephalomyelitis and one associated with a first presentation of multiple sclerosis.

b The NS-SEC three class categorisation was rated at admission and based on the occupation of the patient.

c The most common co-morbidities in those that completed the SF-36 were: hypertension (n = 11), hypercholesterolaemia (n = 5), asthma (n = 5) and thyroid disorder (n = 5). The most common co-morbidities in those that did not complete the SF-36 were: HIV co-infection (n = 9), hypertension (n = 7) and all cause cancer (n = 5).

IQR = interquartile range, NS-SEC = National Statistics Socio-Economic Classification.

A definitive aetiological agent was identified in 11 (55%) of the 20 children for whom the SF-10 was successfully completed. Seven (63.6%) had immune-mediated encephalitis (all ADEM) and 4 (36.4%) had infectious encephalitis (2 bacterial, 1 HSV, 1 VZV). SF-10 non-respondents were less likely to be white British (p = 0.04) than those for which the SF-10 was completed (Table 3).

**Table 3:** Sample Characteristics in those Eligible for the SF-10.

SF-10	Completed the SF-10 (N = 20)	Did not complete the SF-10 (N = 10)	p
**Age, median (IQR), y**	10.0 (8.0–11.0)	10.0 (7.5–11.0)	0.882
**No. (%) male**	**10 (50.0)**	**9 (90.0)**	**0.049**
**Ethnicity, n (%)**			
White British or White Other	**18 (90.0)**	**4 (40.0)**	**0.007**
Asian or Asian British	2 (10.0)	3 (30.0)	0.300
African-Caribbean or Black British	0 (0.0)	2 (20.0)	0.103
Other (inc. Chinese and Mixed Ethnicity)	0 (0.0)	1 (10.0)	0.333
**NS-SEC Three Class Categorisation at admission, n (%)** a			
Higher managerial and professional occupations	3 (15.0)	2 (20.0)	1.000
Intermediate occupations	7 (35.0)	2 (20.0)	0.675
Routine and manual occupations	0 (0.0)	2 (20.0)	0.103
Never worked or long-term unemployed	0 (0.0)	0 (0.0)	1.000
Not classifiable (retired, students, etc)	10 (50.0)	4 (40.0)	0.709
**No. (%) immunocompromised**	2 (10.0)	2 (20.0)	0.584
**No. (%) with at least one co-morbidity**	6 (30.0)	5 (50.0)	0.425
**Glasgow Outcome State 6 months after discharge, n (%)**			
Good recovery	14 (70.0)	6 (60.0)	0.690
Moderate disability	1 (5.0)	3 (30.0)	0.095
Severe disability	5 (25.0)	1 (10.0)	0.633
Dead	0 (0.0)	0 (0.0)	1.000
**Length of stay, median (IQR), d**	21.0 (10.0–39.0)	24.0 (17.5–66.5)	0.677

a The NS-SEC three class categorisation was rated at admission and based on the highest occupational group of the principal guardians.

IQR = interquartile range, NS-SEC = National Statistics Socio-Economic Classification.

### Sf-36 Scores

**Figure 2:**
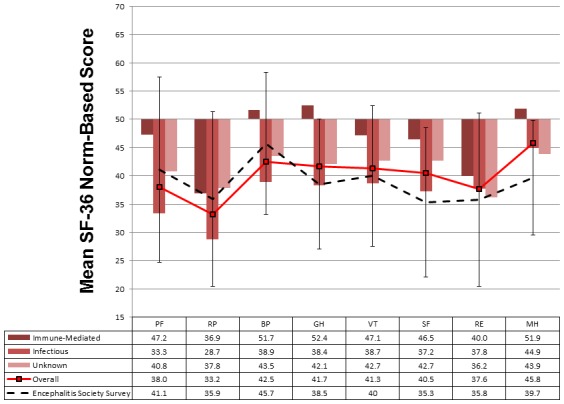
Mean norm-based SF-36 scores for patients after encephalitis compared to the age-and sex-matched general UK population. Scores have been transformed so that the general population scores a mean of 50, with a standard deviation of 10 in each domain; accordingly any score less than 50 is worse than that for the general population. Comparison is made between different aetiological categories and with a survey conducted by the Encephalitis Society in the UK of their adult members with previous self-reported encephalitis [15]. The vertical error whiskers represent the standard deviation for the overall scores for each domain. PF = Physical functioning, RP = role limitation caused by physical dysfunction, BP = bodily pain, GH = general health perceptions, VT = vitality, SF = social functioning, RE = role limitations caused by emotional difficulties, MH = mental health perceptions.

The post-encephalitic population had a poorer HRQoL in all domains of the SF-36 compared to an age- and sex-matched general population (Figure 2). Results were not significantly different after excluding from the analysis the 12 individuals matched to the nearest age group. Our results closely matched those of a survey conducted by the Encephalitis Society of their adult members a median of 10.2 years after a self-reported diagnosis of encephalitis (Figure 2) [15]. The HRQoL following infectious encephalitis was worse than immune-mediated encephalitis in all domains, with the average score being 10 points less than the general population in all except mental health perceptions. Domain scores for patients with encephalitis of unknown cause fell in between the infectious and immune-mediated encephalitis groups. There was large variation in individual scores as suggested by the standard deviation.

### Multivariate Analyses (For The Sf-36)

**Figure 3:**
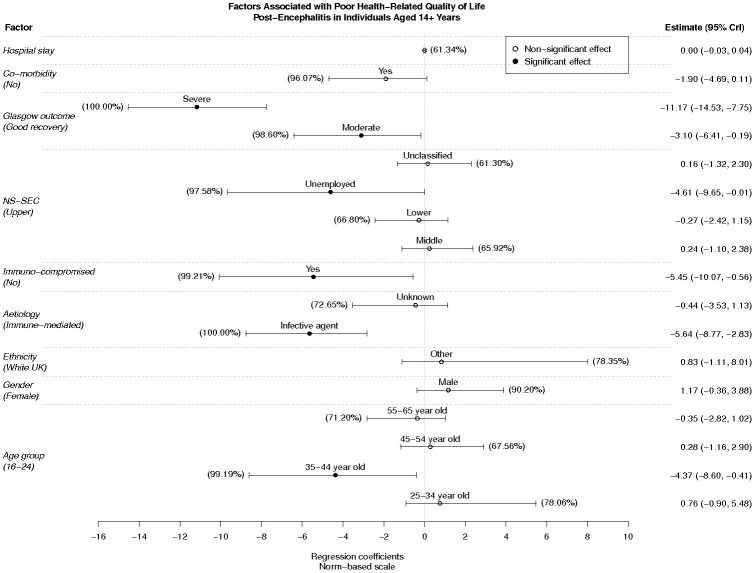
Caterpillar Plot of Estimated Regression Coefficients on Mean Post-encephalitis HRQoL norm based scores. Associated factors are listed along the left axis, with the reference characteristic quoted within parentheses as appropriate. Point estimates (circles) and 95% credibility intervals (whiskers) of each regression coefficient are enumerated along the right axis. Thus, having a co-morbid illness is expected to reduce the norm-based SF-36 score by 1.9 points averaged across all domains, (95% credibility interval −4.69 –0.11 points) compared to those with no co-morbidity. As per Bayesian analysis the percent figures by each whisker indicate the posterior probability of the corresponding regression coefficient being greater or less than zero: the closer the percent value for a given parameter to 100% the greater the portion of its posterior probability mass lies to one side of zero (equivalent to an indication of statistical significance); conversely values closer to 50% indicate proximity to an equal split of the posterior distribution between positive and negative values (indicating a lack of statistical significance).

Inferences on model parameters indicated having infective encephalitis, being immuno-compromised, being unemployed at point of admission, being 35–44 years-old at diagnosis or not showing good recovery six months after discharge all have a significant negative bearing on HRQoL independent of other factors (Figure 3). For instance, a patient who survived infectious encephalitis is expected to exhibit an SF-36 NBS score 5.64 points (95% CI = 2.83–8.77 points) averaged across all domains below that of similar patients recovered from immune-mediated encephalitis. Outcome score as defined by the GOS had the greatest association with negative HRQoL.

### Comparison With Gos And Aetiology

As outcome on GOS deteriorated, the proportions of patients reporting good HRQoL decreased and those reporting a bad HRQoL increased (Table 4). There was a concordant and significant association between the proportion of people reporting a good, moderate or bad HRQoL in the physical functioning (p 0.040), vitality (p 0.030), social functioning (p 0.004) and role emotional (p 0.009) domains of the SF-36 and the proportion assessed as having a good, moderate or bad outcome on the GOS.

**Table 4:** Comparison of proportions of patients reporting a good, moderate and bad HRQoL on the SF-36 with aetiological category and with level of recovery as assessed by the Glasgow Outcome Score (GOS).

	SF-36		Aetiological Category				Glasgow Outcome Score			
		Infectious (N = 31)	Immune-mediated (N = 10)	Unknown (N = 20)	p	Good (N = 29)	Moderate (N = 21)	Severe (N = 11)	p	Total
	Good, n (%)	8 (25.8)	6 (60.0)	8 (40.0)		14 (48.3)	7 (33.3)	1 (9.1)		22 (36.1)
**PF**	Moderate, n (%)	6 (19.4)	1 (10.0)	4 (20.0)	0.414	7 (24.1)	3 (14.3)	1 (9.10	**0.040**	11 (18.0)
	Bad, n (%)	17 (54.8)	3 (30.)	8 (40.0)		8 (27.6)	11 (52.4)	9 (81.8)		28 (45.9)
	Good, n (%)	6 (13.8)	4 (40.0)	8 (40.0)		11 (37.9)	4 (21.1)	1 (9.1)		16 (27.1)
**RP**	Moderate, n (%)	2 (6.9)	1 (10.0)	3 (15.0)	0.094	3 (10.3)	3 (15.8)	0 (0.0)	0.197	6 (10.2)
	Bad, n (%)	23 (79.3)	5 (50.0)	9 (45.0)		15 (51.7)	12 (63.1)	10 (90.9)		37 (62.7)
	Good, n (%)	7 (22.6)	7 (70.0)	8 (40.0)		13 (44.8)	6 (28.6)	3 (27.3)		22 (36.1)
**BP**	Moderate, n (%)	9 (29.0)	1 (10.0)	5 (25.0)	0.129	8 (27.6)	5 (23.8)	2 (18.1)	0.496	15 (24.6)
	Bad, n (%)	15 (48.4)	2 (20.0)	7 (35.0)		8 (27.6)	10 (47.6)	6 (54.6)		24 (39.3)
	Good, n (%)	7 (22.6)	7 (70.0)	6 (33.3)		10 (37.0)	8 (40.0)	2 (18.1)		20 (34.5)
**GH**	Moderate, n (%)	9 (29.0)	2 (20.0)	5 (27.8)	**0.026**	11 (40.8)	3 (15.0)	2 (18.1)	0.094	16 (27.6)
	Bad, n (%)	15 (48.4)	0 (0.0)	7 (38.9)		6 (22.2)	9 (45.0)	7 (63.8)		22 (37.9)
	Good, n (%)	10 (32.2)	6 (60.0)	7 (38.9)		14 (50.0)	8 (40.0)	1 (9.1)		23 (38.5)
**VT**	Moderate, n (%)	6 (19.4)	1 (10.0)	6 (33.3)	0.371	8 (28.6)	1 (10.0	4 (27.3)	**0.030**	13 (22.0)
	Bad, n (%)	15 (48.4)	3 (30.0)	5 (27.8)		6 (21.4)	10 (50.0)	7 (63.8)		23 (38.5)
	Good, n (%)	9 (29.0)	5 (50.0)	10 (50.0)		13 (44.8)	10 (47.6)	1 (9.1)		24 (39.4)
**SF**	Moderate, n (%)	6 (19.4)	4 (40.0)	3 (15.0)	0.108	10 (34.5)	1 (4.8)	2 (18.1)	**0.004**	13 (21.2)
	Bad, n (%)	16 (51.6)	1 (10.0)	7 (35.0)		6 (20.7)	10 (47.6)	8 (72.8)		24 (39.4)
	Good, n (%)	10 (33.3)	2 (20.0)	8 (40.0)		15 (51.7)	5 (25.0)	0 (0.0)		20 (33.3)
**RE**	Moderate, n (%)	8 (25.8)	4 (40.0)	3 (15.0)	0.785	6 (20.7)	5 (25.0)	2 (18.1)	**0.008**	14 (23.4)
	Bad, n (%)	13 (41.9)	4 (40.0)	9 (45.0)		8 (27.6)	10 (50.0)	9 (81.8)		26 (43.3)
	Good, n (%)	15 (48.4)	6 (60.0)	6 (33.3)		15 (53.5)	9 (45.0)	3 (27.3)		27 (45.8)
**MH**	Moderate, n (%)	8 (25.8)	4 (40.0)	6 (33.3)	0.266	8 (28.6)	8 (40.0)	2 (18.1)	0.155	18 (30.5)
	Bad, n (%)	8 (25.8)	0 (0.0)	6 (33.3)		5 (17.9)	3 (15.0)	6 (54.6)		14 (23.7)

The p-values indicate whether the differences in proportions between good, moderate and bad HRQoL in each aetiological category and for each GOS outcome (as calculated through a 3×3 contingency table) are statistically significant (bold) or not. This indicates the strength of the statistical association between each HRQoL domain on one hand, and separately aetiological category and GOS on the other.

PF = Physical Functioning, RP = Role Physical, BP = Bodily Pain, GH = General Health, VT = Vitality, SF = Social Functioning, RE = Role Emotional, MH = Mental Health perceptions.

With regard to aetiology, although the proportions reporting a good HRQoL after immune-mediated encephalitis was much higher than after infectious encephalitis and vice versa in all domains of the SF-36, the difference in proportions was only significant for the general health domain (p 0.026). The proportions reporting good or bad HRQoL after encephalitis of unknown cause were between those of immune-mediated and infectious encephalitis. Generally, few people reported a moderate HRQoL, with the greater proportions at the extremities.

### Sf-10 Scores

**Figure 4:**
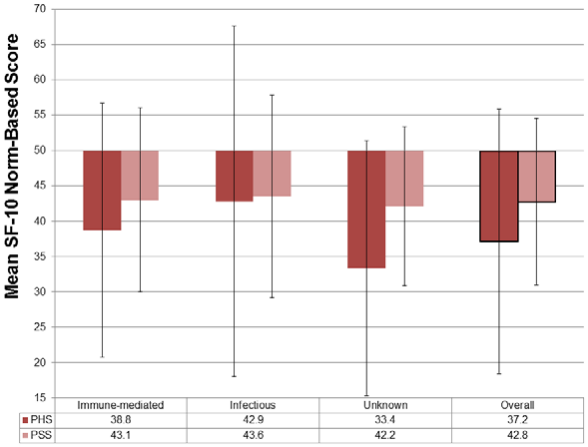
SF-10 scores for a Post-encephalitic Population aged 5–14 years compared with an equivalent US Population Norm [7]. It was necessary to use US-based data for comparison as no equivalent UK normative dataset exists. Scores have been transformed so that the general population scores a mean of 50, with a standard deviation of 10 in each domain; so that any score less than 50 is worse than that for the general population. The vertical bars represent the standard deviation. PHS = physical summary scale, PSS = psychosocial summary scale.

The children for whom the SF-10 was completed had a generally poor HRQoL compared to the age- and sex-matched US general population. Similar to the adult population that completed the SF-36 physical outcomes were more greatly impacted than psychosocial measures and there was variation in scores. (Figure 4). In contrast to the SF-36, the group that had recovered from encephalitis of unknown cause had the worst outcomes.

## Discussion

This study provides compelling evidence that in addition to the significant mortality and morbidity associated with encephalitis, the illness has long-term adverse effects on quality of life for the majority of survivors. Mean scores were consistently reported to be worse in all domains of the SF-36 and SF-10 compared to the general population mean. Overall, HRQoL after encephalitis was in the poor or low-moderate range. Apart from in mental health perceptions, only 25–40% of patients assessed to have made a good recovery on the GOS reported a good HRQoL in the domains of the SF-36 (Table 4).

Infectious encephalitis was associated with the worst quality of life in adults, and encephalitis of unknown cause was associated with the worst quality of life in children, emphasising the need to identify aetiology early in both populations. Physical functioning was more severely impacted than mental health domains in both instruments. As a cohort, patients who responded were less ill and more affluent than those who were eligible but did not respond, indicating the results of this study may under-estimate the overall impact of encephalitis on HRQoL.

There was great spread in the mean scores, which might be explained by the observation that individual scores appeared to aggregate either in the ‘good’ or ‘bad’ range, with only a small proportion reporting a moderate HRQoL after encephalitis (Table 4); suggesting there are significant gains to be made both pre and post diagnosis. This seems particularly true of immune-mediated encephalitis. We have previously shown that antibody-associated encephalitis (a type of immune-mediated encephalitis, the other major type being acute disseminated encephalomyelitis) is associated with the greatest morbidity and mortality [2]. In this study immune-mediated encephalitis was associated with a significantly longer hospital stay. Both these observations may be because encephalitis is difficult to diagnose and immune-mediated encephalitis is particularly under-detected [16], [17]. Although not directly measured, the delay to diagnosis, and thus treatment, may explain the longer hospital stay as well as the significantly poorer outcomes associated with immune-mediated encephalitis; particularly in 2005–8 when patients were enrolled into our study. Yet those who survived tended to report a quality of life equivalent to that of the general population mean, in contrast to those who survived infectious encephalitis.

Several factors were associated with poor outcome overall. Being unemployed at the point of admission and being in the 35–44 year age group affected post-encephalitis HRQoL almost as much as aetiology or immunocompromise. There are a number of potential explanations. We do not have any measure of HRQoL for patients before they became ill. Although unemployment appeared to be associated with worse outcomes, the presence of significant confounding factors cannot be excluded and the correlation between employment at point of admission and post-discharge was not quantified. Regarding the effect of age on HRQoL, the worse outcomes in those aged 35–44 years may be because these individuals are at the peak of their financial and/or family lives, and many may also have young children to provide for (whereas the older age groups are more likely to have adult children as a source of support). An illness such as encephalitis therefore affects these individuals at their greatest level of socio-occupational functioning, and hence their perception of HRQoL may become negatively skewed. Similar effects have been demonstrated after stroke [18].

The strongest association with a fall in HRQoL (as measured by the SF-36) six months after encephalitis was found to be aetiology and GOS. We conducted further analyses to determine the association between these factors and the separate domains of the SF-36. The differences in the proportions reporting good, moderate or poor outcomes and aetiological category were only significant in the General Health domain (Table 4). This domain quantifies how individuals perceive their personal health and whether they think it is likely to improve or deteriorate, suggesting that this may be one factor driving the difference in quality of life after immune-mediated, unknown and infectious encephalitis.

The strongest determinant of HRQoL was GOS outcome so measures to improve GOS such as prompt diagnosis and treatment may have a great impact on post-encephalitic quality of life. In almost all domains, as GOS outcome worsened, proportions of patients reporting a good HRQoL decreased and those reporting a poor HRQoL increased. This was significant for the physical functioning, vitality, social functioning and role emotional domains, indicating that limitations in performing everyday physical activities such as bathing and dressing, and interference with social activities particularly from emotional problems and fatigue may be driving poor outcomes. Targeting these areas for post-encephalitis rehabilitation and support could achieve most gain.

### Limitations

The sample size was not as large as other studies using validated tools to measure HRQoL, particularly for the younger age group. Encephalitis is rare and is associated with high morbidity and mortality; thus many patients will be excluded from an already small sample size if they cannot self-complete the SF-36, for example due to severe disability. There were differences in background characteristics and aetiologies among those who completed the SF- 10 compared with non-respondents and detailed analysis was difficult in this group. Although we were able to follow up >70% of the sample, there was a relatively high rate of attrition in those aged ≥15 years, with only 61 successfully completed SF-36 forms returned from the 118 eligible. A significant proportion of this non-response (35%) could be explained by mortality and severe morbidity. Non-respondents were more likely to be immunocompromised or have another co-morbid illness in both children and adults, and thus it is likely the population not captured by our study have even worse subjective quality of life compared to those that we were able to measure.

Although we only measured HRQoL a median of 5.6 months after discharge from hospital, our results closely matched those of a similar study performed in members of the Encephalitis Society a median of 10.2 years after diagnosis suggesting that HRQoL remains poor in the long-term [15]. We are unable to comment however on the relative impact of the socio-clinical factors we measured on HRQoL in the long-term.

### Strengths

This study represents one of the most comprehensive attempts to measure the quality of life in a post-encephalitic population. It is the largest study to use a standardised case definition to identify encephalitis, and is the first to delineate quality of life by aetiology or measure HRQoL in children. Two highly validated instruments were used to measure the quality of life in individuals aged ≥5 years in an age-appropriate manner, with multiple clinical and socio-demographic factors taken into consideration. This is the most thorough attempt to reconcile the biological causes of encephalitis with its psycho-social impact of which we are aware.

## Conclusions

The mortality and morbidity associated with encephalitis have long been recognised as particularly grave. This study provides the first evidence of the variable quality of life of those who recover and the socio-clinical factors associated with good and bad outcomes. Aetiology and GOS outcome appear to be the two factors most associated with post-encephalitic quality of life, highlighting the need to distinguish aetiology at presentation, particularly as many causes can be treated effectively [19], [20], and the need to identify those at greatest risk and invest in rehabilitation and support for those with long-term disability.
